# Human Papillomavirus Prevalence in Oropharyngeal Cancer before Vaccine Introduction, United States

**DOI:** 10.3201/eid2005.131311

**Published:** 2014-05

**Authors:** Martin Steinau, Mona Saraiya, Marc T. Goodman, Edward S. Peters, Meg Watson, Jennifer L. Cleveland, Charles F. Lynch, Edward J. Wilkinson, Brenda Y. Hernandez, Glen Copeland, Maria S. Saber, Claudia Hopenhayn, Youjie Huang, Wendy Cozen, Christopher Lyu, Elizabeth R. Unger, the HPV Typing of Cancers Workgroup

**Affiliations:** Centers for Disease Control and Prevention, Atlanta, Georgia, USA (M. Steinau, M. Saraiya, M. Watson, J.L. Cleveland, E.R. Unger);; Cedars-Sinai Medical Center, Los Angeles, California, USA (M.T. Goodman);; Louisiana State University, New Orleans, Louisiana, USA (E.S. Peters);; University of Iowa, Iowa City, Iowa, USA (C.F. Lynch);; University of Florida, Gainesville, Florida, USA (E.J. Wilkinson);; University of Hawaii, Honolulu, Hawaii, USA (B.Y. Hernandez);; Michigan Cancer Surveillance Program, Lansing, Michigan, USA (G. Copeland);; Los Angeles Cancer Registry, Los Angeles, California, USA (M.S. Saber, W. Cozen);; University of Kentucky, Lexington, Kentucky, USA (C. Hopenhayn);; Florida Department of Health, Tallahassee, Florida, USA (Y. Huang);; Battelle Memorial Institute, Durham, North Carolina, USA (C. Lyu)

**Keywords:** oropharynx, oropharyngeal, cancer, HPV typing, human papillomavirus, archived tissue, viruses, United States

## Abstract

We conducted a study to determine prevalence of HPV types in oropharyngeal cancers in the United States and establish a prevaccine baseline for monitoring the impact of vaccination. HPV DNA was extracted from tumor tissue samples from patients in whom cancer was diagnosed during 1995–2005. The samples were obtained from cancer registries and Residual Tissue Repository Program sites in the United States. HPV was detected and typed by using PCR reverse line blot assays. Among 557 invasive oropharyngeal squamous cell carcinomas, 72% were positive for HPV and 62% for vaccine types HPV16 or 18. Prevalence of HPV-16/18 was lower in women (53%) than in men (66%), and lower in non-Hispanic Black patients (31%) than in other racial/ethnic groups (68%–80%). Results indicate that vaccines could prevent most oropharyngeal cancers in the United States, but their effect may vary by demographic variables.

Oropharyngeal cancers include malignancies that occur where the oral cavity and pharynx merge, including in the palatine and lingual tonsils, the posterior 1/3 (base) of the tongue, the soft palate, and the posterior pharyngeal wall. Current worldwide incidence has been estimated at ≈85,000 annually (*1*), although it varies extensively by geographic region. In the United States, ≈12,000 new oropharyngeal cancers are diagnosed annually (*2*); most are classified histologically as squamous cell carcinoma (OPSCC). In addition to tobacco use and alcohol use, infection with human papillomavirus (HPV) has been recognized as an independent risk factor for oropharyngeal cancer (*3*–*6*).

The 2 HPV vaccines approved by the US Food and Drug Administration protect against infection with HPV-16 and HPV-18, which are the high-risk types most frequently associated with cervical cancer. A candidate 9-valent vaccine that includes types in the existing quadrivalent vaccine (HPV types 6, 11, 16, and 18) and 5 additional high-risk types (31, 33, 45, 52, and 58) is in clinical trial. Supported by evidence that existing vaccines effectively reduce oral HPV infections, these formulations may also reduce incidence of oropharyngeal cancers (*7*). When monitoring the population-level effect of HPV vaccination on oropharyngeal cancer occurrence in the United States, data on the incidence and type-specific prevalence of this disease are essential. Previously, the prevalence of cases attributable to viral infection and the consequent effects of vaccine programs were approximated from small published studies (*8*–*13*), which estimated HPV to be detected in 37%–60% of OPSCC in North America. Considering the range of prevalence, the heterogeneity of study populations, and differences in sample preparation and HPV detection methods used in these studies, it is not clear that this range of estimates reflects the true scope of HPV-associated OPSCC in the United States. Therefore, the objectives of this study were to determine prevalence of HPV types detected in oropharyngeal cancers in the United States and to establish a prevaccine baseline for monitoring the impact of vaccination. 

## Materials and Methods

### Cancer Tissue Specimens 

As part of the Centers for Disease Control Cancer Registry Sentinel Surveillance System study (M. Saraiya, unpub data), a systematic review of cases of oropharyngeal cancer diagnosed during 1995–2005 was performed. The cases were selected from 7 participating registries, including 4 central cancer registries in Florida, Kentucky, Louisiana, and Michigan and 3 Surveillance, Epidemiology, and End Results program (SEER [http://seer.cancer.gov/] ) cancer registry-based residual tissue repositories in Los Angeles County, CA; Hawaii; and Iowa. The following anatomic regions (by ICD-O-3 codes) were included: C01.9 and C02.4 (base of the tongue and lingual tonsil); C09.0, C09.1, C09.8, C09.9, and c14.2 (tonsil); C14.0, C14.2, C14.8, C02.8, C10.2, C10.8, and C10.9 (other oropharynx) (*13*). Of 4,073 cases matching these criteria, we requested samples from 1,271 case-patients representative of the whole case-patient population regarding sex, age, and race/ethnicity. One archived, formalin–fixed paraffin-embedded tissue sample, representative of the primary tumor, was selected by the submitting pathology laboratory. If tissue from the primary tumor was unavailable, a sample from a metastatic lesion in a lymph node was accepted because HPV prevalence is usually maintained in OPSCC–positive lymph nodes (*14*). With the exception of 32 cases from Hawaii and 11 from Los Angeles County, which had been sampled by Chaturvedi et al. (*12*), cases were selected exclusively for this study. Each participating state and CDC received approval from their institutional review boards for the study; CDC approved the overall study.

### DNA Extraction and HPV Typing

All laboratory methods were described previously (*15*,*16*). Six consecutive 5-μm sections were cut from each selected tissue block; special precautions were used to avoid cross-contamination. The first and last sections were stained with hematoxylin and eosin and reviewed by a study pathologist (ERU) to confirm the presence of viable tumor tissue. DNA was extracted from two 5-μm sections by using high temperature–assisted tissue lysis (*17*) and further purification was carried out by automated extraction by using Chemagic MSM1 (PerkinElmer, Waltham, MA, USA). HPV types were determined from 2 commercial assays by using an algorithm which was evaluated earlier for this application (*18*,*19*). First, all DNA extracts were tested by using the Linear Array HPV Genotyping Assay (Linear Array; Roche Diagnostics, Indianapolis, IN, USA) and a HPV-52-specific PCR to resolve ambiguous positive results from the XR probe of the Linear Array HPV test (*20*). Samples that had negative or inadequate linear array results (negative for HPV and cellular β-globin controls) were retested with the INNO-LiPA HPV Genotyping Assay (Innogenetics, Gent, Belgium). HPV status was recorded for 40 types: 6, 11, 16, 18, 26, 31, 33, 35, 39, 40, 42, 43, 44, 45, 51, 52, 53, 54, 55, 56, 58, 59, 61, 62, 64, 66, 67, 68, 69, 70, 71, 72, 73, 74, 81, 82, 83, 84, IS39, and 89 as tested; and HPV of an unknown type (HPV-X) for additional unspecified types as indicated by LiPA results.

### Analysis

Prevalence was assessed as percentage positive from the total number of cases with valid results. HPV types 16, 18, 31, 33, 35, 39, 45, 51, 52, 56, 58, 59, 66, and 68 were considered to have a high risk for oncogenic potential (*21*) and all other types, including HPV-X, to have a low risk, showing low or no known oncogenic potential.

Hierarchical categories for HPV status were assigned as follows: HPV-16 includes all cases positive for this type regardless of other results. HPV-18 includes all cases positive for HPV-18, but not for HPV-16; other 9-valent, high-risk HPV types included in the next generation of the HPV vaccine: HPV-31, -33, -45, -52, -58, but not HPV-16 or -18; other high-risk HPV cases positive for any high risk not included in the previous categories: HPV-35, -39, -51, -66, -68; and low-risk HPV: all other cases positive for any remaining low-risk HPV types.

Statistical analysis was restricted to case-patients that had confirmed invasive OPSCC. Case-patient age at diagnosis was stratified into 4 groups: <50, 50– 59, 60–69, and >70 years. Cancer stages were crudely classified as local, regional, or distant (metastatic) by SEER classifications. Differences in prevalence of positive results for high-risk HPV or HPV-16/18, categorized by patient’s age, sex, race/ethnicity, and the anatomic location of cancer, were evaluated by using the χ^2^ or Fisher exact test whenever possible. Multivariate analysis was performed by using logistic regression with a step-down procedure, adjusting for age, sex, and race/ethnicity as appropriate. All statistical calculations were made by using SAS 9.3 (SAS Institute Inc., Cary, NC, USA).

## Results

Of the 1,271 oropharyngeal tumors requested from the participating cancer registries, samples from 588 case-patients were received and successfully tested. Those not received were either unavailable or the remaining tissue was not representative of disease. The demographic characteristics (sex, age) and cancer stage (Table 1) (*22*) of the cohort from which the tested sample set was collected were similar to those of the untested cohort. Persons from the Asian Pacific Islands were few in number and slightly overrepresented in the final test population.

**Table 1:** Characteristics of oropharyngeal cancer case-patients who provided samples with those all eligible case-patients, United States, 1995–2005*

Characteristic	% Case-patient samples not tested, n = 683	% Case-patient samples tested, n = 588
Age, y		
<50	18.4	18.5
50–59	32.1	33.7
60–69	27.5	27.6
≥70	22.0	20.2
Race/ethnicity		
Asian/Pacific Islander	1.3	4.4
Non-Hispanic Black	11.9	12.6
Hispanic	8.6	6.8
Non-Hispanic White	77.6	75.2
Other	0.6	1.0
Sex		
F	23.4	25.4
M	76.6	74.5
Cancer stage		
Local	13.6	19.2
Regional	52.0	56.5
Distant	17.7	13.6
Unknown	16.7	10.7
*Samples were provided from 7 US registries.		

HPV results for 476 (81.0%) samples were from the linear array and 112 (19%) from LiPA. Most tissue was from the primary site (n = 473), but for 15 samples, only metastatic tissue from lymph nodes was available. Most case-patients (77.6%) were from urban areas or counties with a population >250,000. Most (94.4%) diagnoses were made during 2000 or later. Median age at the time of diagnosis was 58 (range 28–97) years. The male-to-female ratio was 3:1 and most of the cases (75.6%) were in non-Hispanic White persons. SCC, the most common histologic type of oropharyngeal cancer, accounted for 557 (94.7%) of all cases, and the main analysis was restricted to these cases (Table 2).

**Table 2:** High-risk and HPV types16 and 18 in oropharyngeal squamous cell carcinomas by demographic and tumor characteristics, select United States registries, 1995–2005*

Characteristic	Total no.	High-risk HPV, no. (%) pos.†	HPV-16/18, no. (%) pos.‡
Registry			
Los Angeles Co., CA	20	17 (85.0)	14 (70.0)
Florida	140	101 (72.1)	89 (63.6)
Hawaii	39	33 (84.6)	32 (82.1)
Iowa	13	4 (30.7)	4 (30.7)
Kentucky	116	74 (63.8)	69 (59.5)
Louisiana	95	75 (78.9)	61 (64.2)
Michigan	134	92 (68.6)	79 (59.0)
p value	NA	<0.001	0.032
Age, y			
<50	106	83 (78.3)	74 (69.8)
50–59	191	142 (74.3)	127 (66.5)
60–69	156	102 (65.4)	89 (57.1)
≥70	104	69 (65.4)	58 (55.8)
p value	NA	0.064	0.053
Sex			
F	141	91 (64.5)	74 (52.5)
M	416	305 (74.3)	274 (65.9)
p value	NA	0.053	0.006
Race/ethnicity			
Asian/Pacific Islander	20	16 (80.0)	16 (80.0)
Non-Hispanic Black	71	36 (50.7)	22 (31.0)
Hispanic	39	29 (74.4)	27 (69.2)
Non-Hispanic White	421	310 (73.6)	278 (67.5)
Other	6	5 (83.3)	5 (83.3)
p value	NA	0.002	<0.001
Tumor stage			
Local	102	60 (58.8)	51 (50.0)
Regional	318	248 (78.0)	225 (70.8)
Distant (metastatic)	76	51 (67.1)	42 (55.3)
Unknown	61	37 (60.7)	30 (49.2)
p value	NA	0.001	<0.001
Tumor subsite			
Base of tongue	213	149 (70.0)	129 (60.6)
Tonsil	250	201 (80.4)	181 (72.4)
Other	94	46 (48.9)	38 (40.4)
p value	NA	<0.001	<0.001

*p values for differences between categories were calculated by χ^2^/Fisher exact test. HPV, human papilloma virus; pos., positive; Co., county; NA, not applicable. †Positive for any of the high-risk types: HPV-16, -18, -31, -33, -35, -39, -45, -51, -52, -56, -58, -59, -66, -68. ‡HPV-16/18 = positive for HPV-16 and/or HPV-18.

HPV was detected in 403 of the 557 OPSCC cases (72.4%) with valid typing results and 396 (71.1%) were positive for >1 high-risk type (Table 3). In 68.4% of cases, a single HPV type was found; 3.9% contained 2 types. In 7 cases, only low-risk HPV types were detected: HPV-11, 26, 69, 82 (2 cases), 83, and HPV-X). HPV-16 was present in 337 (60.5%) cases, HPV-18 in 14 (2.5%) cases, and 331 (59.4%) cases were exclusively positive for these 2 types. 

**Table 3:** Human papillomavirus prevalence in oropharyngeal squamous cell carcinomas, select United States registries, 1995–2005*

Variable	No. (%) cases, N = 557
Characteristic	
HPV (any type)	403 (72.4)
High risk†	396 (71.1)
Low risk‡	7 (1.3)
Negative	154 (27.6)
Single type	381 (68.4)
Multiple types§	22 (3.9)
Type	
HPV-16	337 (60.5)
HPV-33	31 (5.6)
HPV-18	14 (2.5)
HPV-35	11 (2.0)
HPV-39	5 (0.9)
HPV-31	4 (0.7)
HPV-52	4 (0.7)
HPV-45	3 (0.5)
Other HPV types	16 (2.9)

*HPV, human papillomavirus; high risk, virus type is associated with high oncogenic potential; low risk, virus type is associated with low oncogenic potential. †Positive for any of HPV types 16, 18, 31, 33, 35, 39, 45, 51, 52, 56, 58, 59, 66, and 68. ‡Positive for types other than those identified as high risk. §HPV-16/-33 (6 cases), HPV-16/-18 (3 cases), and HPV-16/-31(3 cases).

Other high-risk types, including HPV-31, 33, 35, 39, 45, and 52, were found at low frequency (Table 3). The relative prevalence in case-patients that had multiple HPV types essentially followed single–type distributions. HPV-16/33 was the most frequent combination (6 cases); HPV-16/18 and HPV-16/31 were the next most frequent, found in samples from 3 case-patients each. Frequencies of all co-detected HPV types are shown in Table 4. More than 2 types were not found in any of the oropharyngeal cancers.

**Table 4:** Oropharyngeal squamous cell carcinomas with >1 HPV type, select US registries, 1995–2005*

Type combination	No. cases
HPV-16 and -33	6
HPV-16 and -18	3
HPV-16 and -31	3
HPV-16 and -35	2
HPV-16 and -45	1
HPV-16 and -52	1
HPV-16 and -54	1
HPV-16 and -59	1
HPV-16 and -83	1
HPV-18 and -35	1
HPV-33 and -39	1
HPV-39 and -56	1

*No cases with >2 types were found; HPV, human papillomavirus.

Proportions of high-risk HPV prevalence and HPV-16/18 were statistically different among the registries and by race/ethnicity, stage, and anatomic subsite (Table 2). By sex, prevalence was only different for those infected with HPV-16/18. Age at diagnosis was not statistically different between the stratified groups, but median age at diagnosis among high-risk HPV positive case-patients was 58 (28–92) years and 61 (36–97) years in high-risk negative case-patients (p = 0.023).

**Figure 1:**
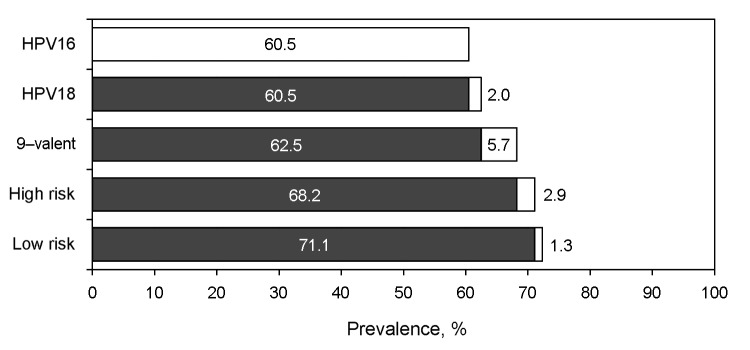
Hierarchical designation of human papillomavirus (HPV) types to oropharyngeal squamous cell carcinomas. White sections of bars indicate attribution of the specific HPV type or group. Black sections of bars indicate cumulative prevalence of types in higher hierarchy. HPV-16 includes all cases positive for this type regardless of other results. HPV-18 includes all cases positive for HPV-18, but negative for HPV-16. Cases of 9-valent HPV with high-risk HPV types included in the candidate 9-valent HPV vaccine: HPV-31, -33, -45, -52, -58, but not HPV-16 or -18. High-risk: cases positive for any high-risk type not included in the previous categories: HPV-35, -39, -51, -66, -68. Low-risk: cases only positive for HPV types with low or no oncogenic potential.

According to hierarchical assessment, HPV-16 was found in 337 (60.5%) OPSCC cases, HPV-18 in 11 (2.0%), other 9-valent high-risk types in 32 (5.7%), other high-risk types in 16 (2.9%), and low-risk types in the remaining 7 (1.3%) cases (Figure 1). Of the 15 case-patients for whom lymph node metastases were tested, 14 were positive for high-risk HPV and 13 were positive for HPV-16.

In multivariate analysis for high-risk HPV that included age and sex in the model, only race/ethnicity was a significant independent factor (p = 0.003). Odds for high-risk HPV infections were significantly higher for all other race groups than for non-Hispanic Black persons (p<0.001). When only HPV-16/18 detection was considered, significant differences were found in sex (p = 0.009) and race/ethnicity in (p<0.001), but not age (p = 0.063), between those infected and those who were not (Table 5).

**Table 5:** Multivariate analysis for HPV and 18 detection in 557 oropharyngeal squamous cell carcinoma samples, select United States registries, 1995–2005*

Variable	OR (95% CI)†	p value
Sex		0.009
F	Ref	
M	1.70 (1.14–2.55)	
Race/ethnicity		<0.001
Non-Hispanic Black	Ref	–
Asian/Pacific Islander	8.43 (2.51–28.29)	–
Hispanic	4.73 (2.02–11.1)	–
Non-Hispanic White	4.34 (1.17–97.47)	–

*HPV, human papillomavirus; OR, odds ratio; Ref, reference group.  †Odds ratio adjusted for race and ethnicity; age was not significant.

The 31 cases that had histologic results other than SSC included 7 adenocarcinomas (2 were HPV-16 positive, 5 HPV negative) and 2 small cell or neuroendocrine carcinomas (both HPV negative). Twenty-two cases were carcinomas not further specified, of which 7 tested positive for HPV (4 for HPV-16, and 1 each for HPV-18, HPV-33, HPV-35).

## Discussion

Our finding of >70% HPV prevalence in a large sample of the oropharyngeal cancer patients from around the United States suggests that a substantially higher faction may be HPV-related than has been reported in many previous investigations (*14*). In a systematic review of HPV prevalence studies, including several small investigations of populations in North America, Kreimer et al. estimated that 47% of OPSCC cases were HPV related (*11*). Chaturvedi et al. more recently estimated weighted HPV prevalence at 72% (*12*), which is comparable to our findings. A continuous increase in HPV-related OPSCC that was observed during the past 20 years and escalated after 2000 (*23*) may explain some of the discrepancies found in the literature. More sensitive laboratory methods may also have contributed to the increased HPV prevalence relative to earlier investigations. All studies in North America considered by Kreimer et al. (*11*) relied on the MY09/11 or GP5+/6+ consensus primer sets that require intact HPV L1 fragments of 450 and 150 bp, respectively. By contrast, in the study by Chaturvedi et al. (*12*) and ours, testing incorporated the INNO-LiPA assay, which has SFP primers that target 65-bp amplicons. Assays targeting smaller L1 amplicons can achieve increased sensitivity for HPV detection in formalin-fixed, paraffin-embedded tissues known to have smaller DNA templates in their extracts than fresh or frozen samples do (*18*). Although not identical in specificity to that of the Linear Array, INNO-LiPA had shown comparable performance for detecting single type HPV infections predominantly found in cancer tissues (*19*).

Our current study results further confirm a trend of increasing incidence of tonsillar cancers with shifting demographic patterns (*24*,*25*).The results further confirmed that HPV-16, detected in 84% of all positive tissues, was by far the most frequent type found in oropharyngeal cancers. Although type 16 also has nominally the highest prevalence in the “normal” oral cavity or oropharynx, other types are usually found at similar frequency (*26*,*27*). The ability of HPV-16 to establish persistent infection and its potential to transform might be responsible for its prominence in cancers. Currently available HPV vaccines targeting HPV-16 and -18 may be highly effective against OPSCC (*9*). A candidate 9-valent vaccine (currently in clinical trials) could have the potential to prevent virtually all HPV-associated oropharyngeal cancers: our data showed that 2.9% of the case-patients were positive for a high-risk type not covered in this formulation. (Figure 1).

The most noticeable differences were observed between racial groups, with notably fewer HPV-positive SCCs in non-Hispanic Black persons (50.7%) compared with non-Hispanic White persons (73.6%), Hispanic persons (74.4%), or Asian Pacific Islanders (80.0%). Other studies that noted similar differences by race/ethnicity found this to be a recent but ongoing development (*28*). Settle et al. (*29*), who investigated oral cancer survival, also reported reduced HPV prevalence in Black persons compared with other race/ethnicity groups and found that this difference was particular to oropharyngeal cancer and not to other cancers of the oral cavity.

In addition to differences by race/ethnicity, HPV prevalence also varied by sex, particularly for HPV-16/18. Prevalence was 66% among men, notably higher than the 53% found among women, which was a finding consistent with results of other investigations (*2*). Although further data stratification might show even greater dissimilarities, for instance between Black women and White men, the sample sizes for these analyses were modest and confidence intervals were large (data not shown). The precise causes for these discrepancies are unknown and most likely complex, but may be anticipated to influence vaccine efficacy for OPSCC. Prevalence differences observed between the registry states may be, in part, caused by demographic variations. Difference in age at diagnosis between patients with HPV-positive and HPV-negative cases was borderline significant (p = 0.023). Although other studies have also shown that HPV-positive cancers occur in a younger population (*30*), the role of 3 years difference in median age is unclear. It is possible that differences in behavior associated with causal pathways, such as smoking and drinking, provide a partial explanation. Persistent HPV infection at these anatomic sites may occur early, leading to more rapid and damaging alterations in cell cycle regulation and proliferation than those that occur with other carcinogenic exposures.

Of particular note, high-risk HPV types were detected in 80% of tonsillar SCCs. The microanatomy of the lymphoepithelial tissue of Waldeyer’s ring, most notably the lingual and palatine tonsils, may explain this finding. Deep invaginations in this area by the tonsillar crypts may expose immature basal cells to HPV (*31*). The median age of case-patients was slightly lower than that of those with cancer in other sites (55 years), but proportions of infection, when sex and race/ethnicity were considered, were not different than proportions for the other oropharyngeal sites (data not shown). One limitation of this study is that not all participating sites were able to perform systematic random sampling of case-patient specimens from their eligible pool. The sizable specimen collections from the 4 cancer registries (Michigan, Kentucky, Louisiana, and Florida) were sampled by a simple random or systematic sampling approach, on the basis of the number of eligible cases. Sampling from the SEER tissue repositories (Los Angeles, California; Hawaii; and Iowa) was dependent on what tissue specimens were available. However, the resulting sample population that was ultimately tested represented diverse geographic regions and a wide range of demographic variables regarding sex, age, and race/ethnicity.

It should be noted that the presence of HPV DNA does not confirm its causal role in carcinogenesis. Detection in tumor tissues potentially overestimates the true involvement of the virus because coincidental, transient infections and complementary transforming effects to other factors cannot be distinguished. The natural history of cases in this study could not be assessed in this retrospective cross-sectional study and behavioral data were not available from the participating cancer registries. In particular, information regarding tobacco or alcohol use and HIV status would potentially improve estimation of the proportion of OPSCC caused by HPV alone. Because it is not clear at this point if HPV alone is sufficient to cause oropharyngeal cancer, factors other than use of tobacco products should be considered. Additional HPV markers, such as viral transcription (particularly E6 and E7 mRNA) or characteristic gene expression profiling, may provide further insights in future assessments and show distinction between actively transforming HPV infections and random, transient occurrences (*32*,*33*). Similar investigations may also be warranted to explicate the 7 cases in which only low-risk types were found. It is likely that these HPV types were present coincidentally and played no role in malignant transformation, but genomic changes that altered their pathogenic properties to bring them closer to those of high-risk types could provide an intriguing alternative explanation.

## Conclusions

This study supports a role for oncogenic HPV in high proportions of oropharyngeal cancers. Future assessments are needed to monitor general prevalence and possible type-specific shifts. Data from the present and future studies will provide a baseline for early assessment of vaccine effects. Because the natural history and pre-cancer stages of oropharyngeal cancers are not established as they are for cervical cancer, direct trials with oropharyngeal neoplasia as the endpoint are not feasible. To obtain meaningful, comparable data for this objective, researchers need a universal definition of the anatomic oropharynx and associated malignancies and agreement on laboratory methods.
